# Antibacterial Activity of *Aristolochia brevipes * against Multidrug-Resistant *Mycobacterium tuberculosis*

**DOI:** 10.3390/molecules16097357

**Published:** 2011-08-29

**Authors:** Víctor Manuel Navarro-García, Julieta Luna-Herrera, Ma. Gabriela Rojas-Bribiesca, Patricia Álvarez-Fitz, María Yolanda Ríos

**Affiliations:** 1 Laboratory of Microbiology, Biomedical Research Center of the South (IMSS), Argentina 1, Col. Centro, 62790 Xochitepec, Morelos, Mexico; 2 Immunochemistry Laboratory II, Department of Immunology, National School of Sciences Bilogy (IPN), Prolongación Carpio y Plan de Ayala S/N, Col. Casco de Santo Tomás, 11340 Mexico, D.F., Mexico; 3 Chemical Research Center, University of Morelos (UAEM), Av. Universidad 1001, Col. Chamilpa, 62209 Cuernavaca, Morelos, Mexico

**Keywords:** *Mycobacterium tuberculosis*, Multidrug-resistant (MDR), *Aristolochia brevipes*, alamarBlue™ microassay, medicinal plant

## Abstract

The increased incidence of Multidrug-Resistant *Mycobacterium tuberculosis* (MDR-MT) requires the search for alternative antimycobacterial drugs. The main aim of this study was to evaluate the dichloromethane extract from *Aristolochia brevipes* (Rhizoma) and the compounds isolated from this extract against several mycobacterial strains, sensitive, resistant (monoresistant), and clinical isolates (multidrug-resistant), using the alamarBlue™ microassay. The extract was fractionated by column chromatography, yielding the following eight major compounds: (1) 6α-7-dehydro-*N*-formylnornantenine; (2) *E/Z*-*N*-formylnornantenine; (3) 7,9-dimethoxytariacuripyrone; (4) 9-methoxy-tariacuripyrone; (5) aristololactam I; (6) β-sitosterol; (7) stigmasterol; and (8) 3-hydroxy-α-terpineol. The structures of these compounds were elucidated by ^1^H- and ^13^C- (1D and 2D) Nuclear Magnetic Resonance (NMR) spectroscopy. This study demonstrates that the dichloromethane extract (rhizome) of *A. brevipes* possesses strong *in vitro* antimycobacterial activity against *Mycobacterium tuberculosis* H37Rv (Minimum Inhibitory Concentration value [MIC], 12.5 µg/mL). The most active compound against all mycobacterial strains tested was the compound aristolactam I (**5**), with MIC values ranging between 12.5 and 25 µg/mL. To our knowledge, this the first report of antimycobacterial activity in this plant.

## 1. Introduction

*Mycobacterium tuberculosis* (MTB) is one of the species of the so-called tuberculosis complex and is the causative agent of tuberculosis (TB). Pulmonary tuberculosis is transmitted from person-to- person by means of secretions of the throat and lungs of persons presenting active respiratory disease. According to the World Health Organization (WHO), in 2008 there were 9.4 million cases worldwide, of which 3.6 million were females, among whom 500,000 patients presented co-infection with the Human Immunodeficiency Virus (HIV), and there were 1.8 million deaths, with an average of 4,500 deaths daily, due to this disease. In this same year, the worldwide incidence of the disease diminished to 139 cases per 100,000 inhabitants, after which in 2004 there have been 143 cases per 100,000 inhabitants [1]. In Mexico, 2005, 14,443 cases of tuberculosis were registered, with an incidence of 13.7/100,000 inhabitants [2]. Diverse studies have demonstrated that one of every 10 infected individuals eventually developed some form of TB [3].

The increase in the number of cases of TB has been associated with the infection of humans with HIV, in addition to the appearance and development of TB-resistant drugs, both multidrug-resistant (MDR), as well as extremely drug-resistant (XDR). Drug-resistant TB is caused by non-compliance with the treatment period, by prescription of an inadequate treatment regimen, by administration of inadequate drugs, *etc.* Multidrug-resistant MDR-TB is a potentially deadly disease caused by the bacilli of isoniazid (INH)-resistant TB and rifampicin (RIF)-resistant TB. Treatment for MDR-TB requires the use of at least three antibiotics, generally a combination of first- and second-line antibiotics; XDR-TB presents when, in addition to first line-drug resistance, resistance to second-line drugs is present, rendering treatment of these patients very complicated. Unfortunately, the success of curing cases of MDR-TB and XDR-TB is very low [4].

The appearance of mycobacteria that are resistant to the drugs most frequently utilized for treatment signals the need for increasing therapeutic research, basing the search on novel molecules with antimycobacterial properties. The challenge of discovering of new, urgently needed anti-TB drugs has led to the need for new antimicrobial compounds that have diverse chemical structures and novel mechanisms of action. Natural products or their semisynthetic derivatives, provide novel examples of such anti-infective drugs [5]. Natural products are a proven template for the development of new scaffolds for drug [6,7]; and these have received considerable attention as potential anti-TB agents. In Mexico and in many cities of the world, there has been a great surge in the study of medicinal plants, with the purpose of knowing the chemical characteristics of their secondary metabolites and their biological activity. In this work, we studied the antimycobacterial activity of dichloromethane extract and some constituents of the Mexican plant *Aristolochia brevipes*, popularly known as “guaco”. This is a plant that grows in several states of the Mexican Republic, such as Michoacán, Colima, Guerrero, and Morelos, among others. Persons mainly use the rhizomes to treat arthritis, diarrhea, to cleanse wounds, and for cough with blood, and also to cure serpent wounds [8,9]. We prepared a dichloromethane extract from which eight compounds were obtained, but we only tested the following: 6α-7-dehydro-*N*-formylnornantenine (**1**); *E/Z*-*N*-formylnornantenine (**2**); 7,9-dimethoxytariacuripyrone (**3**); 9-methoxytariacuripyrone (**4**) and aristololactam I (**5**), all of which were previously isolated from *A. brevipes* [10,11]; however, there are no reports on pharmacological activity, specifically antimycobacterial; thus, to our knowledge, this is reported here for the first time.

## 2. Results and Discussion

**Figure 1:**
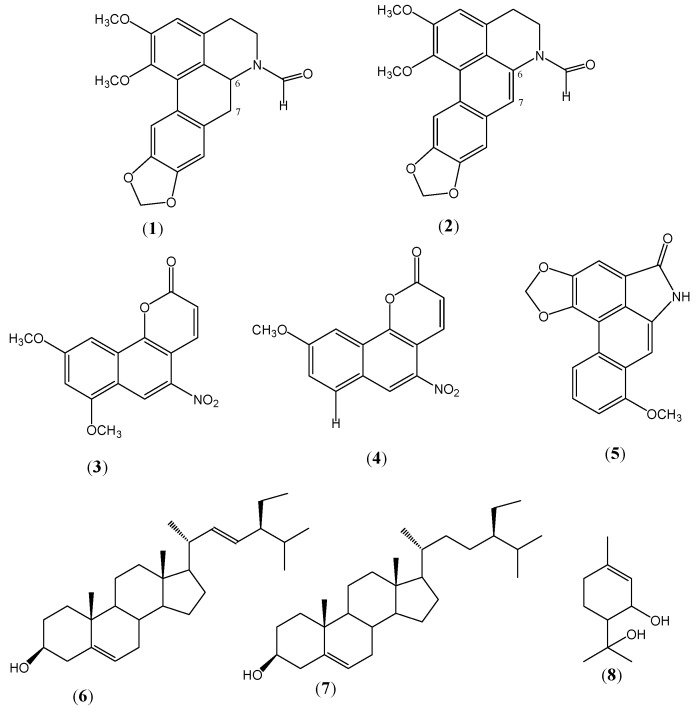
Structure of the compounds from *Aristolochia brevipes*.

We determined the antimycobacterial activity of the *A. brevipes* of the dichloromethane extract utilizing a reference strain sensitive to *Mycobacterium tuberculosis* first-line antibiotics (H37Rv), obtaining important activity, with a MIC value of 12.5 µg/mL. This extract was submitted to fractionation through conventional chromatographic methods, which allowed for isolation of the following eight compounds: 6α-7-dehydro-N-formyl-nornantenine (**1**) [10]; *E/Z*-*N*-formylnornantenine (**2**) [12]; 7,9-dimethoxytariacuripyrone (**3**) [10]; 9-methoyitariacuripyrone (**4**) [10]; aristololactam I (**5**) [13]; stigmasterol (**6**); β-sitosterol (**7**); and 3-hydroxy-α-terpineol (**8**) (Figure 1). The structures of all of the compounds obtained were established unequivocally through analysis of their spectroscopic data of ^1^H- and ^13^C-NMR and by comparison of these with those published in the literature [10,11,12,13].

Compounds **1–5** were evaluated against four monoresistant variants and three MDR clinical isolates of *Mycobacterium tuberculosis*; the results of their activity are presented in Table 1.

**Table 1:** Antimycobacterial activity (Minimum inhibitory concentration [MIC] µg/mL) of the compounds isolated from *Aristolochia brevipes*.

Compound	(1) ^b^	(2)	(3)	(4)	(5)	(6)
Microorganism						
H37Rv ^a^	>50 ^c^	>50	25.0	25.0	12.5	12.5
H37RvEr	>50	>50	50.0	25.0	12.5	NT ^d^
H37RvIr	>50	>50	12.5	50.0	25.0	NT
H37RvRr	>50	>50	50.0	50.0	25.0	NT
H37RvSr	>50	>50	25.0	25.0	25.0	NT
MMDO	>50	NT	50.0	50.0	25.0	NT
MTY147	>50	NT	50.0	25.0	12.5	NT
SIN452	>50	NT	25.0	25.0	12.5	NT

^a^ H37Rv: drug-sensitive *Mycobacterium tuberculosis*. H37RvEr: etambutol-resistant *M. tuberculosis*. H37RvIr: isoniazid-resistant *M. tuberculosis*. H37RvRr: rifampicin-resistant *M. tuberculosis*. H37RvSr: streptomycin-resistant *M. tuberculosis*. MMDO: clinically isolated etambutol and isoniazid-resistant. MTY147: clinically isolated isoniazid and rifampicin-resistant. Sin452: clinical isolate rifampicin, isoniazid, and etambutol-resistant; ^b^ (**1**) 6α-7-dehydro-*N*-formylnornantenine; (**2**) *E/Z-N*-formylnornantenine; (**3**) 7-9-dimethoxytariacuripyrone; (**4**) 9-methoxytariacuripyrone; (**5**) aristololactame I; (**6**) total extract (dichloromethane); ^c^ >50 was considered as non-active; ^d^ NT; Not tested.

We found no report in the literature on the antituberculous evaluation of these compounds, specifically in compounds **1–5**; thus, this report constitutes, to our knowledge, the first report on this activity. Compounds **1** and **2** are benzyltetrahydroisoquinoline alkaloids, which did not demonstrate antituberculosis activity in this study (Table 1). Other alkaloids of this type also have been isolated in *Aristolochia constricita*, and these demonstrated a significant decrease in the electrically induced contraction, with acetylcholine and guinea pig ileum-isolated histamine [14], while compounds **3** and **4** are 5-nitro-2*H*-benzo[h]chromenones (presumably derived from aristolochic acid) [15], popularly known as “tariacuripironas”. To our knowledge, there are no reports in the literature on biological evaluations of this type of compounds; we only found the description of strong mutagenic activity for 9-methoxy-tariacuripyrone (**4**) against TA98, TA100, and YG of *Salmonella typhimurium* strains [16]. With regard to the antituberculous activity of these compounds, *i.e.*, **3** and **4**, they demonstrated very good antituberculous activity against all of the assayed strains (sensitive strain, monoresistant, and clinically isolated, MDR), with MIC values ranging between 25 and 50 µg/mL, with the exception of *Mycobacterium tuberculosis* H37RvIr, for which we obtained an MIC value of 12.5 µg/mL for 7–9 methoxytariacuripyrone (**3**) (Table 1).

The compound that demonstrated the greatest inhibitory activity against all of the *Mycobacterium* strains assayed, antibiotic- sensitive, monoresistant variants, and clinically isolated multidrug resistant spp. (MDR) was aristolactam (**5**), whose MIC values were found to range from 12.5 to 25.0 µg/mL (Table 1). This compound is an aristolactam-type alkaloid and it has been confirmed that its presence is not exclusive to the Aristolochiaceae family; rather, this type of compound has also been found in *Stephania cepharantha* (Menispermaceae) [17] and in *Schefferomitra subaequalis* (Annonaceae) [18]. In 1995 Tian-Shung [19] determined the cytotoxic activity of aristolactam (**5**) and other aristolactams. The majority of the compounds assayed demonstrated significant cytotoxicity against the KB, P-388, A-549, HT-29, and HL-60 cell lines. Later, it was reported that compound **5** forms adducts in assays with DNA [20]; thus, this product it of great interest as an anticancer agent, and now with the important results of anti-tuberculosis activity reported here, may be useful for the development of anti-tuberculosis drugs.

The active compounds found in this study require greater chemical and pharmacological studies, due to that they exhibit a certain cytotoxocity and mutagenicity, but it also true that currently, new molecules are required that combat the aggressivity of MDR-TB. Therefore, in this work, we present some molecules that are active against this type of resistant bacteria.

## 3. Experimental

### 3.1. Collection of Plant Material

The root of *Aristolochia brevipes* was collected from its natural habitat in the municipality of Xochitepec in the Mexican state of Morelos during the months of September and October, 2008. Herbolarium specimens were prepared, which were deposited for classification at the Cuernavaca City, Morelos, National Institute of Anthropology and History Herbolarium by Margarita Avilés, M.Sc., and Macrina Fuentes, M.Sc. (INAHM-2033).

### 3.2. General Procedure and Equipment Utilized

The compounds were purified by means of Open Column Chromatography (OCC) on Merck Kiesegel 60. The isolation procedure and the purity of the compounds obtained were monitored by Thin Layer Chromatography (TLC) (Silica gel 60 F_254_ Merck, Germany), visualized by ultraviolet light (UV), and revealed with ceric ammonium sulfate (Sigma Chemical Co., St. Louis, MO, USA). Infrared (IR) spectra were obtained in CHCl_3_ on a Bruker Vector 22 infrared instrument. Nuclear magnetic resonance (NMR) experiments (^1^H, and ^13^C) were obtained at 400 MHz for ^1^H and at 100 MHz for ^13^C in CDCl_3_ on Varian Unity 400 equipment, utilizing tetramethylsilane (TMS) as internal reference.

### 3.3. Extract Preparation, Separation, and Isolation of Secondary Metabolites

The extract of the dry ground root of *A. brevipes* (1.5 kg) was obtained by maceration with dichloromethane (3 L, 24 h × 3). The macerated material was filtered and evaporated under low pressure in a rotatory evaporator. The extract obtained in this manner (28 g) was submitted to successive chromatographic processes by OCC. This procedure was initiated with 100% *n*-hexane as eluent system, and polarity was gradually increased by means of successive additions of acetone to obtain five groups of fractions: G1 (0.800 g); G2 (3.4 g); G3 (9.0 g); G4 (0.35 g), and G5 (3.0 g), which were the result of combining the fractions obtained according to their homogeneity and the characteristics observed by TLC. Elution of the G1 group of fractions with *n*-hexane-acetone (9.5:0.5), provided a mixture of the sterols β-sitosterol and stigmasterol (0.42 g, 1.5% with respect to the extract dry weight). The G2 group, eluted with *n*-hexane-acetone (9:1), permitted the isolation of 6α-7-dehydro-*N*-formyl-nornantenine (**1**, 0.99 g, 3.5%) as the major compound. Group G3, eluted with *n*-hexane-acetone (9:1), allowed furnished **1**, on this occasion as the minor component and the compounds 9-methoxytariacutipyrone (**2**, 2.03 g, 7.2%) and 7,9-dimethoxytariacuripirone (**3**, 5.4 g, 19.5%) as the major compounds. The G4 group, eluted with *n*-hexane-acetone (7:3), gave **2** and aristololactam I (**4**) (0.24 g, 0.84%). Finally, the G5 group was eluted with the *n*-hexane-acetone mixture (6:4) and allowed the isolation of *E/Z-N*-formylnornantenine (**5**, 1.5 g, 5.3%) and 3-hydroxy-α-terpineol (0.078 g, 0.27%). The latter compound and sterols were not tested in this study.

### 3.4. Mycobacterium Strains and Clinical Isolates

The following *Mycobacterium* strains were obtained from the American Type Culture Collection (ATCC): *M. tuberculosis* H37Rv (27294); isoniazid-resistant H37Rv (35822); streptomycin-resistant H37Rv (35820); rifampicin-resistant H37Rv (35838), and etambutol-resistant H37Rv (35837). We employed clinical isolates of *Mycobacterium tuberculosis* obtained from patients under care at different hospitals in Mexico. The clinical isolates were selected based on a drug resistance pattern obtained previously by means of Microplate alamar blue assay (MABA) performed on the following drugs: isoniazid; rifampicin; etambutol; streptomycin; kanamycin; rifabutin; ofloxacin; clarithromycin; clofazimine; ethionamid, and amikacin.

### 3.5. Inoculum Preparation for Biological Assay

The reference strains and clinical isolates were incubated at 37 °C in Middlebrook 7H9 broth (BBL), supplemented with 0.2% glycerol and 10% of OADC enrichment (oleic acid, albumin, dextrose, catalase; Difco) until log-phase growth was achieved. The inoculum for the microcolorimetric assay was prepared by diluting log phase growth cultures with sterile 7H9 to the McFarland No. 1 turbidity standard; after the suspension, we adjusted the work suspension to 1:10.

### 3.6. Antimycobacterial Activity Determination by Fluorometric Microplate Alamar Blue Assay

The methodology was fully described by Luna-Herrera [21], but some modifications were made in the present study. A stock solution of total extract was prepared in DMSO at a concentration of 20 g/L; pure compounds were dissolved in DMSO at a concentration of 5 g/L. The test was performed in 96-well sterile microplates in which sterile water (200 µL) was added to the outer-perimeter wells. All other wells received 7H9 broth (100 µL). Then, a working solution (100 µL) was added to the well. Next, serial two-fold dilutions were prepared and an aliquot of the bacterial suspension (100 µL) was added to these wells. Final testing concentrations are 200–6.25 µg/mL for the extract and from 50–1.56 µg/mL for pure compounds. Simultaneously a 1:10 diluted control was prepared from the bacterial suspension, which represented the growth of 10% of the bacterial population tested. The plates were incubated at 37 °C after 5 days of incubation, one control was developed with 20 µL of alamarBlue™ solution (Trek Diagnostics, Westlake, OH, USA). The plates were reincubated at 37 °C for 24 h after this incubation if the well turned pink, all wells received alamarBlue™ solution and were incubated for an additional 24 h. Fluorescence was measured in a plate fluorometer (Fluoroskan Ascent FL, Thermo, Finland) at a 490-nm excitation wavelength and a 540-nm emission wavelength and Relative fluorescence units (RFU) were recorded. Minimum inhibitory concentration (MIC) was defined as the lowest extract of pure compounds concentration that presented RFU values lower than those presented by the 10% growth control. There was always a correlation between fluorometric and visual observation, *i.e.*, pink wells presented high RFU values.

## 4. Conclusions

The present study supports the fact that organic extract of *Aristolochia brevipes* provides an excellent opportunity to find active molecules against resistant *Mycobacterium tuberculosis* (MDR-T). The crude extract also showed important against *Mycobacterium tuberculosis*; which is related with the use of the plant as an anti-infective agent.
